# Indian Medical Service

**Published:** 1916-09-02

**Authors:** 


### ROYAL NAVAL MEDICAL SERVICE.

(*See page* 506.)

### ROYAL ARMY MEDICAL CORPS.

### INDIAN MEDICAL SERVICE.

I. *Present procedure for appointment.*—It has been announced in the Press that after the open competitive examination held in July 1915 for admission to the Indian Medical Service no similar examination would be held during the continuance of the war, but that such appointments as might be required to meet the absolutely indispensable needs of the Service would be made by nomination by the Secretary of State. To assist him in making these appointments, which, as already announced, will be limited in number to the absolutely indispensable needs of the Service, Mr. Chamberlain has appointed a Selection Committee who will summon and interview such applicants as may appear to be *primâ facie* suitable, and make recommendations for appointment.

Applications for appointment should be addressed to the Military Secretary, India Office, Whitehall, S.W., and should contain concise particulars of the applicant's medical degrees and career. Applicants must be over 21 and under 32 years of age at the time of application. Particulars regarding pay, promotion, etc., in the Service can be obtained from the Military Secretary.

The remarks in the first paragraph of Section II. below apply to candidates for appointment by nomination *except* in regard to the upward limit of age.

II. *Procedure for appointment in force at the last Competitive Examination.*—Candidates must be natural-born subjects of His Majesty, of European or East Indian descent, between 21 and 28 years of age at the date of the examination, of sound bodily health, and in the opinion of the Secretary of State for India in Council in all respects suitable to hold commissions in the Indian Medical Service. They may be married or unmarried. They must possess, under the Medical Acts in force at the time of their appointment, a registrable qualification to practise both medicine and surgery in Great Britain and Ireland.

The physical fitness of each candidate will be determined by a Board of Medical Officers, who are required to certify that his vision is sufficiently good to enable him to pass the tests laid down by the Regulations. The candidate will be examined by the Examining Board in the following subjects : (1) Medicine, including therapeutics; (2) surgery, including diseases of the eye; (3) applied anatomy and physiology; (4) pathology and bacteriology; (5) midwifery and diseases of women and children; (6) materia medica, pharmacology, and toxicology. The examination in medicine and surgery will be in part practical, and will include operations on the dead body, the application of surgical apparatus, and the examination of medical and surgical patients at the bedside. No syllabus is issued in the subjects of the examination, which will be conducted so as to test the general knowledge of the candidate in these subjects. No candidate shall be considered eligible who shall not have obtained at least one-third of the marks obtainable in each of the above subjects and one-half of the aggregate marks for all the subjects.

After passing the competitive examination, the successful candidates will be required to attend a course of practical instruction at the Army Medical School and elsewhere, as may be decided, in : (1) hygiene; (2) military and tropical medicine; (3) military surgery; (4) pathology of diseases and injuries incidental to military and tropical service. This course will be of not less than four months' duration, and at its conclusion candidates will be required to pass an examination in the subjects taught therein.

III. After the war the Secretary of State will make further appointments to the Service from among duly qualified persons, European and Indian, who have held temporary commissions in the Indian Medical Service or the Royal Army Medical Corps during the war, and have served with the British or Indian Expeditionary Forces or hospitals and hospital ships for soldiers. The date of the resumption of competitive examinations, and the conditions of such examinations, will be announced in due course.

Full particulars can be obtained by application to the Military Secretary, India Office, London, S.W.

